# Prognostic significance of distal subtotal gastrectomy with standard D2 and extended D2 lymphadenectomy for locally advanced gastric cancer

**DOI:** 10.1038/srep17273

**Published:** 2015-11-25

**Authors:** Chun-Dong Zhang, Ming-Yang Shen, Jia-Kui Zhang, Fei-Long Ning, Bao-Sen Zhou, Dong-Qiu Dai

**Affiliations:** 1 Department of Gastrointestinal Surgery, the Fourth Affiliated Hospital of China Medical University, Shenyang, Liaoning, China; 2 Department of Epidemiology, School of Public Health, China Medical University, Shenyang, Liaoning, China; 3 Cancer Research Institute, China Medical University, Shenyang, Liaoning, China; 4 Cancer Center, the Fourth Affiliated Hospital of China Medical University, Shenyang, Liaoning, China

## Abstract

This study was conducted to investigate prognosis and survival of patients undergoing distal subtotal gastrectomy with D2 and D2+ lymphadenectomy for patients with locally advanced gastric cancer. Overall survival rates of 416 patients with locally advanced gastric cancer were compared between D2 and D2+ lymphadenectomy. Univariate analysis and multivariate analysis was used to identify significant prognostic factors correlated with LN metastasis and prognosis. Univariate analysis identified tumor size, lymphatic vessel invasion, pT stage, pN stage, TNM stage, locoregional recurrence, and distant recurrence, to significantly correlate with prognosis; Tumor size, LVI, and pT stage were identified as independent factors correlating with LN metastasis. Multivariate analysis demonstrated that tumor size, pT stage, pN stage, locoregional recurrence, and distant recurrence were independent prognostic factors; Tumor size and pT stage were independent prognostic factors predicting LN metastasis. When comparing 5-year survival rates of patients who underwent D2 and D2+ lymphadenectomy, as stratified by pT stage and pN stage, a significant difference was found in pN3 patients, but not for pT2–4 and pN0–2 patients, or the patient cohort as a whole. In conclusion, D2 lymphadenectomy for patients with locally advanced gastric cancer undergoing distal subtotal gastrectomy was recommended, especially in eastern Asia.

Gastric cancer remains a major global public health problem^1^,^2^,^3^. Although the incidence of gastric cancer has been declining, it remains the fifth most frequently diagnosed cancer and the second leading cause of cancer-related death worldwide^4^,^5^. Unfortunately, it is often diagnosed at an advanced stage and is associated with poor survival. Radical surgery remains the only potential curative modality for patients with resected gastric cancer. Remarkably, the incidence of gastric cancer is the highest in China^6^.

The number of metastatic lymph nodes (LNs; pN stage) is considered one of the most reliable prognostic indicators for patients with radically resected gastric cancer. The International Union Against Cancer (UICC)/American Joint Committee on Cancer (AJCC) N staging system depends on an adequate number (≥15) of metastatic LNs retrieved^7^,^8^. The Japanese Gastric Cancer Association (JGCA) N staging system depends on the level of metastatic LNs - at least second-level (D2 lymphadenectomy) for optimal staging^9^. However, both the UICC/AJCC N staging system and the JGCA N staging system depend on ≥15 LNs for optimal staging.

A more extensive LN dissection may contribute to more LNs retrieved, which may also improve staging accuracy. However, its contribution to prolonged survival remains unclear; much of the survival benefit associated with extensive lymphadenectomy may only be due to stage migration (“Will Rogers” effect)^10^,^11^,^12^,^13^. Moreover, more extensive surgery can also contribute to more operation-related complications and mortality. Therefore, the efficacy of various LN dissection levels remains controversial^7^,^11^,^14^,^15^.

Gastrectomy with D1 or modified D2 lymphadenectomy, with a goal of ≥15 LNs retrieved has been recommended for patients with localized resected gastric cancer in western countries^7^,^11^,^15^. Recently, standard D2 lymphadenectomy has become the standard treatment for curable gastric cancer in eastern Asia, especially in Japan and China. However, previous studies have shown that a more extensive LN dissection improves survival in patients with advanced gastric cancer^11^,^12^,^16^. Extended D2 (D2+) lymphadenectomy may help to retrieve more LNs for patients with gastric cancer compared with D2 lymphadenectomy, which may contribute to adequate staging and a beneficial survival outcome. To our knowledge, there is no study comparing survival after D2 and D2+ lymphadenectomy for locally advanced gastric cancer with distal subtotal gastrectomy. In light of these considerations, we aimed to investigate prognosis and survival of patients undergoing distal subtotal gastrectomy with D2 and D2+ lymphadenectomy for patients with locally advanced gastric cancer.

## Patients and Methods

All patients with advanced gastric cancer who underwent surgery in our institution were entered into a retrospectively maintained database between February 1990 and February 2014. A total of 424 patients with locally advanced gastric cancer patients underwent distal subtotal gastrectomy with D2 or D2+ lymphadenectomy, and achieved a potentially curative resection for histologically proven gastric carcinoma. Moreover, patients with pre-operative chemo-radiation or chemotherapy were excluded in this study.

This study was approved by the Ethics Committee of the Fourth Affiliated Hospital, China Medical University. All patient records and information were anonymized and de-identified prior to analysis. The methods were carried out in “accordance” with the approved guidelines. We confirm that informed consent was obtained from all subjects.

The follow-up period of the entire population was complete until death or the cutoff date of October 2014. All patients underwent history and physical examination, and had CEA and CA19–9 levels assessed every 3 to 6 months for the first postoperative year, and every 6 to 12 months thereafter. The median and mean follow-up durations were 36 and 62 months, respectively (range 1–286 months). Eight patients were lost to follow-up and were excluded from this study. The rate of follow-up was 98.1%.

Of the included 416 patients with locally advanced gastric cancer, 287 (69.0%) underwent D2 lymphadenectomy. An average of 18.35 ± 9.72 LNs were retrieved, with 4.92 ± 5.01 metastatic LNs. One-hundred and twenty-nine (31.0%) patients underwent D2+ lymphadenectomy, with an average of 20.80 ± 10.38 retrieved LNs and 5.37 ± 6.86 metastatic LNs.

Only patients with locally advanced gastric cancer in stage II and stage III (pT2–4aN0–3M0: including, T3N0M0, T2N1M0, T4aN0M0, T3N1M0, T2N2M0, T4aN1M0, T3N2M0, T2N3M0, T4aN2M0, T3N3M0, and T4aN3M0) were included in this study (T2, tumor invades muscularis propria; T3, tumor penetrates subserosal connective tissue without invasion of visceral peritoneum or adjacent structures; T4a, invades serosa; N0, no regional LNs metastasis; N1, 1–2 regional LNs metastasis; N2, 3–6 regional LNs metastasis; N3, ≥7 regional LNs metastasis)^8^.

According to the National Comprehensive Cancer Network (NCCN) guidelines for gastric cancer, D2 lymphadenectomy should include regional LNs (perigastric LNs) and those along the named vessels of the celiac axis (left gastric artery, common hepatic artery, celiac artery, splenic hilum, and splenic artery)^8^. For distal subtotal gastrectomy, D2 lymphadenectomy includes No. 1, No. 3, No. 4sb, No. 4d, No. 5, No. 6, No. 7, No. 8a, No. 9, No. 11p, and No. 12a LNs. D2+ lymphadenectomy includes LNs of D2 and one or more of these LNs, including, No. 8p, No. 12b, No. 13, and No. 14v LNs.

D2+ lymphadenectomy was recommended for patients who were highly suspected with level 2 LN metastasis, preoperatively, according to contrast-enhanced computed tomograph (CT) scans of the abdomenm, preoperatively. Moreover, D2+ lymphadenectomy was also recommended for patients who were highly suspected with level 2 LN metastasis, according to the macroscopic appearance of level 2 LNs, intraoperatively. Importantly, D2+ lymphadenectomy was recommended if level 2 LN metastasis was proved by pathology detection, intraoperatively.

Recurrences were classified as locoregional recurrence and distant recurrence. Locoregional recurrence included any cancer recurrence in gastric bed, anastomotic sites, and regional LNs. Distant recurrence included visceral metastases, peritoneal metastases, and LN metastases beyond the regional LNs. Importantly, all recurrences were diagnosed clinically or radiographically, with histopathologic test or radiographic test, including CT of head, chest, abdomen, and pelvis; bone scans, or even positron emission tomography-CT (PET-CT) would be applied if necessary. Moreover, the incidence of each pattern of recurrence was compared between patients with D2 lymphadenectomy and D2+ lymphadenectomy. (Table 1)

The carcinoma lesions together with the surrounding gastric wall were fixed in formalin and cut into multiple 5 mm slices, which were parallel to the lesser curvature; two pathologists independently examined the sections and disagreements were resolved by discussion to determine the final diagnosis. According to the current NCCN guidelines for gastric cancer, examining at least 15 LNs was strongly recommended for adequate staging^7^,^11^. Thus, if fewer than 15 LNs are initially identified, resubmission should be performed in order to identify as many LNs as possible.

Overall survival rates were calculated using Kaplan-Meier survival analysis. An event was defined as death for a cancer-related cause. Two sided χ^2^ tests or two-tailed t-tests were performed for statistical comparison of clinicopathologic features between patients with D2 and D2+ lymphadenectomy. The log-rank test was conducted in the univariate analysis and multivariate analysis was used to identify significant prognostic factors correlated with LN metastasis. Cox’s proportional hazard model was applied to identify significant factors correlating with prognosis, according to the results of the univariate analysis. Scatter diagrams were used for distribution of metastatic LNs and retrieved LNs, between D2 and D2+ lymphadenectomy. A p-value of less than 0.05 was defined as statistically significant. SPSS version 20.0 statistical software was used for statistical analysis (SPSS Inc., Chicago, IL, USA).

## Results

A total of 416 patients with locally advanced gastric cancer who underwent distal subtotal gastrectomy were included in this study. Of these, 287 patients (median age 58.61 ± 10.63 years) underwent D2 lymphadenectomy and 129 patients (median age 57.86 ± 11.07 years) underwent D2+ lymphadenectomy. For patients who underwent D2 lymphadenectomy, 81 (28.2%) were female and 206 (71.8%) were male. For patients who underwent D2+ lymphadenectomy, 48 (37.2%) were female and 81 (62.8%) were male.

**Figure 1:**
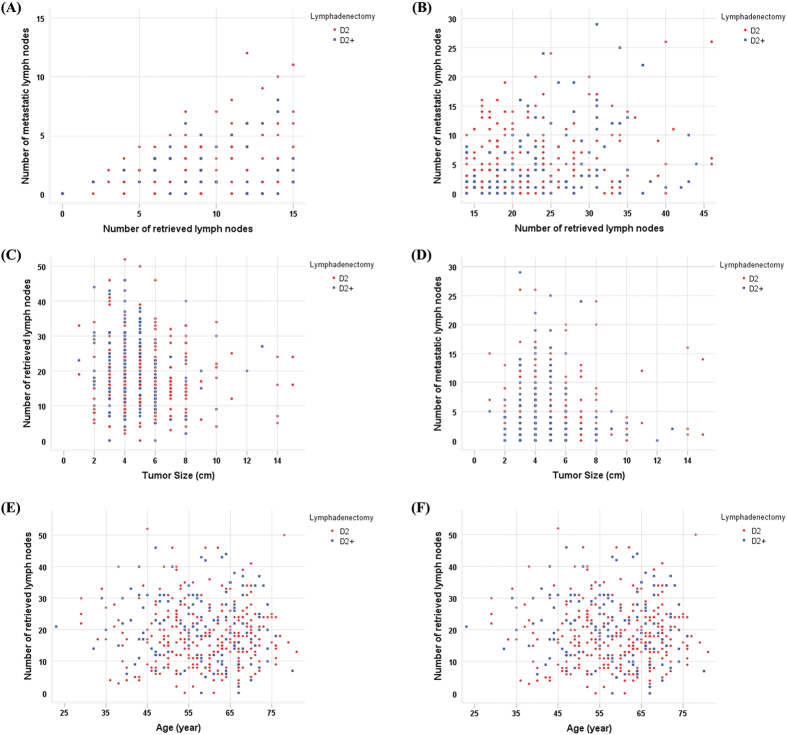
(**A**) The distribution of the number of metastatic LNs according to the number of retrieved LNs for patients with ≤15 LNs retrieved comparing D2 with D2+ lymphadenectomy. (**B**) The distribution of the number of metastatic LNs according to the number of retrieved LNs for patients with >15 LNs retrieved comparing D2 with D2+ lymphadenectomy. (**C**) The distribution of the number of retrieved LNs according to tumor size comparing D2 with D2+ lymphadenectomy. (**D**) The distribution of the number of metastatic LNs according to tumor size of patients comparing D2 with D2+ lymphadenectomy. (**E**) The distribution of the number of retrieved LNs according to age of patients comparing D2 with D2+ lymphadenectomy. (**F**) The distribution of the number of metastatic LNs according to age of patients comparing D2 with D2+ lymphadenectomy.

Clinicopathologic features were comparable between patients undergoing D2 and D2+ lymphadenectomy (Table 1). Significant differences were only found with regards to history (p = 0.044), tumor size (5.24 ± 2.29 versus 4.74 ± 1.97, p = 0.032), number of LNs retrieved (18.35 ± 9.72 versus 20.80 ± 10.38, p = 0.020), and pT stage (p = 0.044). Figure 1 also shows the relationship between the number of metastatic LNs and number of retrieved LNs, number of retrieved LNs and tumor size, number of metastatic LNs and tumor size, number of retrieved LNs and age, and number of metastatic LNs and age.

**Figure 2:**
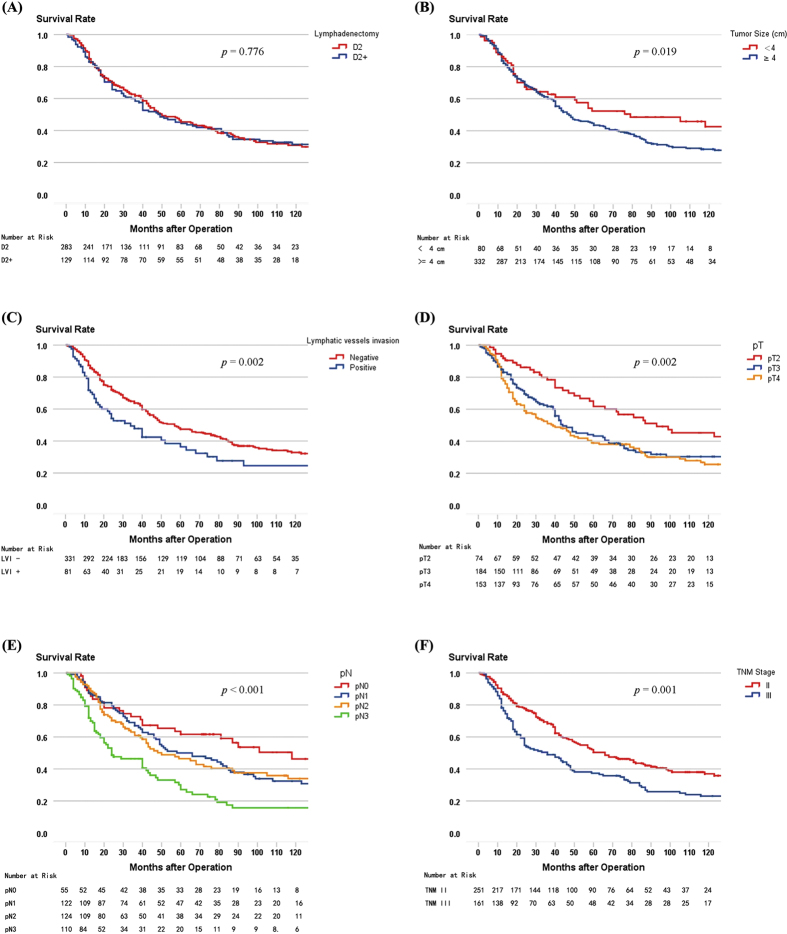
(**A**) Survival curves of patients according to lymphadenectomy (n = 416, p = 0.776). (**B**) Survival curves of patients according to tumor size (n = 416, p = 0.019). (**C**) Survival curves of patients according to status of lymphatic vessels invasion (n = 416, p = 0.002). (**D**) Survival curves of patients according to pT stage (n = 416, p = 0.002). (**E**) Survival curves of patients according to pN stage (n = 416, p < 0.001). (**F**) Survival curves of patients according to TNM stage (n = 416, p = 0.001).

Univariate analysis identified tumor size (p = 0.019), lymphatic vessel invasion (LVI) (p = 0.002), pT stage (p = 0.002), pN stage (p < 0.001), and TNM stage (p = 0.001) to significantly correlate with prognosis for the entire population (Fig. 2). Multivariate analysis demonstrated that tumor size (RR 1.474, 95% CI 1.051–2.065, p = 0.024), pT stage (RR 1.260, 95% CI 1.057–1.502, p = 0.010), pN stage (RR 1.187, 95% CI 1.028–1.370, p = 0.020), locoregional recurrence (RR 2.383, 95% CI 1.545–3.676, p < 0.001), and distant recurrence (RR 2.346, 95% CI 1.563–3.521, p < 0.001) were independent prognostic factors for the entire population; however, lymphatic vessel invasion and TNM stage were not (Table 2).

For the 360 patients with LN metastasis, the univariate analysis identified tumor size (p = 0.021), LVI (p = 0.013), and pT stage (p < 0.001) as independent factors correlating with LN metastasis (Table 3). As shown, the multivariate analysis confirmed that tumor size (RR 1.475, 95% CI 1.035–2.103, p = 0.032) and pT stage (RR 1.444, 95% CI 1.210–1.723, p < 0.001) were independent prognostic factors predicting LN metastasis (Table 3). Figure 2 shows survival curves comparing D2 and D2+ lymphadenectomy, tumor size, LVI, pT stage, pN stage, and TNM stage.

**Figure 3:**
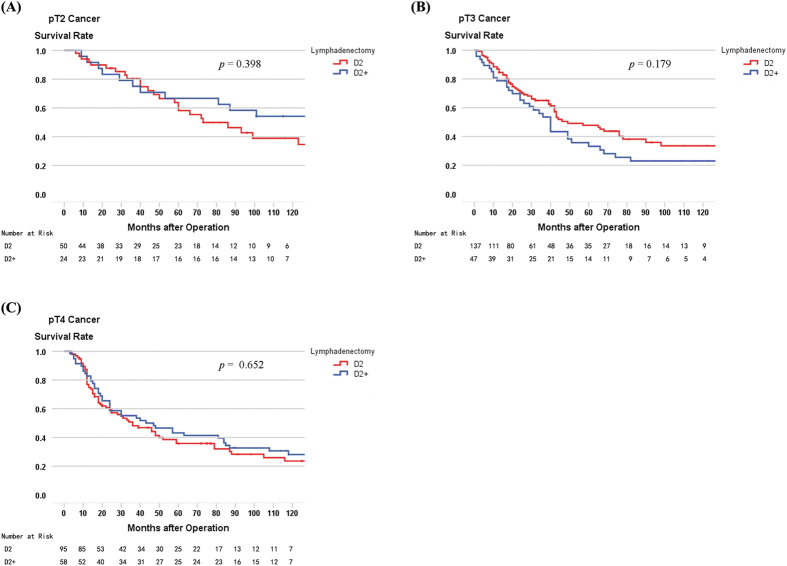
(**A**) Survival curves of pT2 patients according to lymphadenectomy (n = 75, p = 0.398). (**B**) Survival curves of pT3 patients according to lymphadenectomy (n = 187, p = 0.179). (**C**) Survival curves of pT4 patients according to lymphadenectomy (n = 154, p = 0.652).

**Figure 4:**
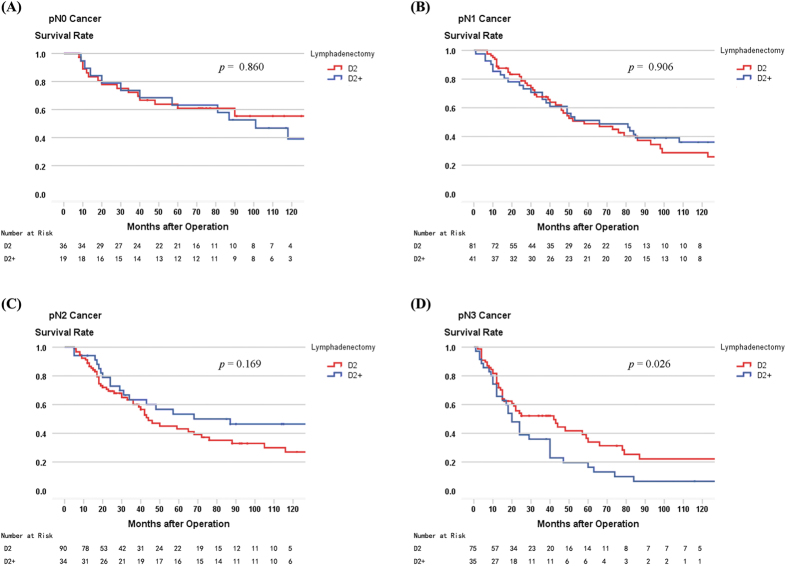
(**A**) Survival curves of pN0 patients according to lymphadenectomy (n = 56, p = 0.860). (**B**) Survival curves of pN1 patients according to lymphadenectomy (n = 122, p = 0.906). (**C**) Survival curves of pN2 patients according to lymphadenectomy (n = 126, p = 0.169). (**D**) Survival curves of pN3 patients according to lymphadenectomy (n = 112, p = 0.026).

When comparing 5-year survival rates of patients who underwent D2 and D2+ lymphadenectomy, as stratified by pT stage and pN stage, a significant difference was found in pN3 patients (33.9% versus 16.3%, log-rank, p = 0.026), but not for pT2–4 and pN0–2 patients, or the patient cohort as a whole (45.4% versus 44.4%, log-rank, p = 0.776; Table 4). The survival curves comparing D2 and D2+ lymphadenectomy in patients with pT2–4 and pN0–3 stages are shown in Figs 3 and 4, respectively.

## Discussion

Radical surgery remains the primary potentially curable treatment for patients with resected gastric cancer. Complete resection with a negative margin (R0 resection) has been regarded as the standard goal and subtotal gastrectomy is preferred for patients with distal gastric cancer, as this approach has similar outcomes with significantly fewer complications as compared to total gastrectomy^17^. Thus, only patients with distal gastric cancer who underwent subtotal gastrectomy were included in this study.

Recently, D2 lymphadenectomy has become the standard treatment for curable gastric cancer in eastern Asia. Moreover, previous research has shown that patients with advanced gastric cancer benefit from a more extensive LN dissection^11^. D2+ lymphadenectomy is known to access more LNs compared with D2 lymphadenectomy, which may contribute to adequate staging and survival benefit. Moreover, D2+ lymphadenectomy has the same morbidity and mortality as D2 lymphadenectomy^18^. Therefore, we conducted this study to investigate survival outcome for patients undergoing distal subtotal gastrectomy with D2+ or D2 lymphadenectomy for locally advanced gastric cancer.

Documentation of insufficient number and level of LNs retrieved is recommended by both the UICC and JGCA. Insufficient LN retrieval leads to under-staging, or “stage migration”^10^. Both the UICC and JGCA N staging systems recommend retrieving a minimum of 15 LNs. If the number of retrieved LNs is inadequate (<15), down-staging may occur with residual positive LNs^19^,^20^. In this study, the median number of retrieved LNs was 18.35 and 20.80 for D2 and D2+ lymphadenectomy, which was a significant difference although both types of lymphadenectomy achieved an adequate number of retrieved LNs (>15 LNs).

We purport that LN metastasis is a poor prognostic factor for patients with gastric cancer and that the number of positive LNs significantly influences survival^21^. In our cohort, the prevalence of LN metastasis was 86.5% (87.1% in patients with D2 lymphadenectomy and 85.3% in patients with D2+ lymphadenectomy). LN metastasis was more frequently observed in patients with a larger tumor size (≥4 cm), LVI, and a greater depth of invasion (pT stage). The multivariate analysis confirmed tumor size and pT stage as independent factors predicting LN metastasis, indicating that larger tumors with a greater depth of invasion had a higher risk of LN metastasis and therefore a worse survival. Thus, we advise that as many LNs should be retrieved as possible to avoid residual LNs, especially for larger tumors with a greater depth of invasion.

We also confirmed that patients with locally advanced gastric cancer failed to benefit from D2+ lymphadenectomy as compared with D2 lymphadenectomy. In order to investigate the independent factors predicting LN metastasis, univariate and multivariate analyses were conducted. No significant difference was observed between D2 and D2+ lymphadenectomy for patients with LNs metastasis, confirming the fact that D2+ failed to decrease the incidence of LNs metastasis.

When comparing 5-year survival between D2 and D2+ lymphadenectomy for locally advanced gastric cancer, we found no significance between D2 and D2+ lymphadenectomy for the entire patient cohort (45.4% versus 44.4%, p = 0.776) and for those with pT2, pT3, pT4, pN0, pN1, and pN2 stages. Importantly, D2 lymphadenectomy was found to have a superior survival rate compared with D2+ lymphadenectomy in pN3 patients. No 5-year survival benefit was found in patients undergoing D2+ lymphadenectomy compared with D2 lymphadenectomy. Therefore, D2+ lymphadenectomy should not be recommended for patients with locally advanced gastric cancer in eastern Asia.

Our study had several limitations. First, this retrospective study was based on 24-year follow-up data, and the time frame may be too large to be able to adequately address the research question, as patients, surgeons, surgical techniques, surgical scales, skills, and adjuvant therapy have changed. Second, we did not compare surgical complications between patients who underwent D2 and D2+ lymphadenectomy. Third, we didn’t include patients with pre-operative chemo-radiation or chemotherapy. The results of our study must be weighed based on these points, which should be clarified in further studies.

In conclusion, we demonstrated that D2+ lymphadenectomy failed to improve survival for patients with locally advanced gastric cancer undergoing distal subtotal gastrectomy, as compared with D2 lymphadenectomy. However, sufficient experience and a high degree of training is required to perform D2 lymphadenectomy. Based on our results, we recommend D2 lymphadenectomy for patients with locally advanced gastric cancer undergoing distal subtotal gastrectomy, especially in eastern Asia.

## Additional Information

**How to cite this article**: Zhang, C.-D. *et al*. Prognostic significance of distal subtotal gastrectomy with standard D2 and extended D2 lymphadenectomy for locally advanced gastric cancer. *Sci. Rep*. **5**, 17273; doi: 10.1038/srep17273 (2015).

## Figures and Tables

**Table 1:** Clinicopathologic features of patients with D2 and D2+ lymphadenectomy (n = 416).

Variables	D2 lymphadenectomy n = 287	D2+ lymphadenectomyn = 129	p value
Sex			0.067
Female	81 (28.2)	48 (37.2)	
Male	206 (71.8)	81 (62.8)	
Age (years)	58.61 ± 10.63	57.86 ± 11.07	0.510
Previous history			0.044
Ulcer	42 (14.6)	21 (16.3)	
Gastritis	23 (8.0)	12 (9.3)	
Gastritis and ulcer	3 (1.1)	7 (5.4)	
None	219 (76.3)	89 (69.0)	
Tumor size (cm)	5.24 ± 2.29	4.74 ± 1.97	0.032
Macroscopic type			0.801
Borrmann 1	7 (2.4)	2 (1.6)	
Borrmann 2	51 (17.8)	19 (14.7)	
Borrmann 3	215 (74.9)	101 (78.3)	
Borrmann 4	14 (4.9)	7 (5.4)	
Histologic grade			0.145
Well differentiated	81 (28.2)	45 (34.9)	
Moderately differentiated	58 (20.2)	28 (21.7)	
Poorly differentiated	140 (48.8)	49 (38.0)	
Undifferentiated	8 (2.8)	7 (5.4)	
Margin status			0.378
Negative	282 (98.3)	125 (96.9)	
Positive	5 (1.7)	4 (3.1)	
Venous invasion			0.901
Negative	283 (98.6)	127 (98.4)	
Positive	4 (1.4)	2 (1.6)	
Lymphatic vessels invasion			0.087
Negative	224 (78.0)	110 (85.3)	
Positive	63 (22.0)	19 (14.7)	
Number of LNs retrieved	18.35 ± 9.72	20.80 ± 10.38	0.020
pT stage			0.044
pT2	51 (17.8)	24 (18.6)	
pT3	140 (48.8)	47 (36.4)	
pT4	96 (33.4)	58 (45.0)	
pN stage			0.667
pN0	37 (12.9)	19 (14.7)	
pN1	81 (28.2)	41 (31.8)	
pN2	92 (32.1)	34 (26.4)	
pN3	77 (26.8)	35 (27.1)	
LN metastasis			0.612
No	37 (12.9)	19 (14.7)	
Yes	250 (87.1)	110 (85.3)	
Number of LNs metastasis	4.92 ± 5.01	5.37 ± 6.86	0.454
TNM stage			0.106
Stage II	182 (63.4)	71 (55.0)	
Stage III	105 (36.6)	58 (45.5)	
Reconstruction type			0.170
Billroth I	231 (80.5)	111 (86.0)	
Billroth II	56 (19.5)	18 (14.0)	
Number of LNs retrieved			0.367
Inadequate (n < 15)	102 (35.5)	40 (31.0)	
Adequate (n ≥ 15)	185 (64.5)	89 (69.0)	
Locoregional recurrence			0.286
Absent	213 (74.2)	102 (79.1)	
Present	74 (25.8)	27 (20.9)	
Distant recurrence			0.404
Absent	186 (64.8)	89 (69.0)	
Present	101 (35.2)	40 (31.0)	
Chemotherapy			0.073
No	193 (67.2)	98 (76.0)	
Yes	94 (32.8)	31 (24.0)	

Two tailed t-tests of mean ± standard deviation (SD); n, number of patients; LNs, lymph nodes.

**Table 2:** Univariate and multivariable analysis of prognostic factors for the entire population (n = 416).

Variables	Univariate analysis		Multivariate analysis		
	n = 416 (%)	p value	RR	95% CI	p value
Sex		0.374			
Female	129 (31.0)				
Male	287 (69.0)				
Age (years)		0.153			
<65	281 (67.5)				
≥65	135 (32.5)				
Tumor size (cm)		0.019	1.474	1.051-2.065	0.024
<4	81 (19.5)				
≥4	335 (80.5)				
Macroscopic type		0.298			
Borrmann 1	9 (2.2)				
Borrmann 2	70 (16.8)				
Borrmann 3	316 (76.0)				
Borrmann 4	21 (5.0)				
Histological grade		0.999			
Well differentiated	126 (30.3)				
Moderately differentiated	86 (20.7)				
Poorly differentiated	189 (45.4)				
Undifferentiated	15 (3.6)				
Venous invasion		0.301			
Negative	410 (98.6)				
Positive	6 (1.4)				
Lymphatic vessels invasion		0.002			
Negative	334 (80.3)				
Positive	82 (19.7)				
Margin status		0.755			
Negative	407 (97.8)				
Positive	9 (2.2)				
pT stage		0.002	1.260	1.057–1.502	0.010
pT2	75 (18.0)				
pT3	187 (45.0)				
pT4	154 (37.0)				
pN stage		<0.001	1.187	1.028–1.370	0.020
pN0	56 (13.5)				
pN1	122 (29.3)				
pN2	126 (30.3)				
pN3	112 (26.9)				
TNM stage		0.001			
Stage II	253 (60.8)				
Stage III	163 (39.2)				
Lymphadenectomy		0.776			
D2	287 (69.0)				
D2+	129 (31.0)				
Number of LNs retrieved		0.578			
Inadequate (n<15)	142 (34.1)				
Adequate (n ≥ 15)	274 (65.9)				
Locoregional recurrence		<0.001	2.383	1.545–3.676	<0.001
Absent	315 (75.7)				
Present	101 (24.3)				
Distant recurrence		<0.001	2.346	1.563–3.521	<0.001
Absent	275 (66.1)				
Present	141 (33.9)				

n, number of patients; LNs, lymph nodes; RR, relative risk; 95% CI, 95% confidence interval.

**Table 3:** Univariate and multivariate analysis of factors predicting LN metastasis (n = 360).

Variables	Univariate analysis			Multivariate analysis		
	LN metastasis (+)	Incidence (%)	p value	RR	95% CI	p value
Sex			0.272			
Female	108	83.7				
Male	252	87.8				
Age (years)			0.087			
<65	243	86.5				
≥65	117	86.7				
Tumor size (cm)			0.021	1.475	1.035–2.103	0.032
<4	71	87.7				
≥4	289	86.3				
Macroscopic type			0.563			
Borrmann 1	9	100				
Borrmann 2	58	82.9				
Borrmann 3	275	87.0				
Borrmann 4	18	85.7				
Histological grade			0.935			
Well differentiated	107	84.9				
Moderately differentiated	73	84.9				
Poorly differentiated	171	90.5				
Undifferentiated	9	60.0				
Venous invasion			0.730			
Negative	355	86.6				
Positive	5	83.3				
Lymphatic vessels invasion			0.013			
Negative	284	85.0				
Positive	76	92.7				
Margin status			0.951			
Negative	352	86.5				
Positive	8	88.9				
pT stage			<0.001	1.444	1.210-1.723	<0.001
pT2	70	93.3				
pT3	175	93.6				
pT4	115	74.7				
Number of LNs retrieved			0.563			
Inadequate (n < 15)	114	80.3				
Adequate (n ≥ 15)	246	89.8				
Lymphadenectomy			0.812			
D2	250	87.1				
D2+	110	85.3				

n, number of patients; LN, lymph node; RR, relative risk; 95% CI, 95% confidence interval.

**Table 4:** Comparison of overall survival outcomes among patients with D2 and D2+ lymphadenectomy stratifying by pT and pN stage (n = 416).

Variables	D2 lymphadenectomy		D2+ lymphadenectomy		p value
	n = 287	5-YSR %	n = 129	5-YSR %	
Entire population	287	45.4	129	44.4	0.776
pT stage					
pT2	51	58.2	24	66.7	0.398
pT3	140	47.8	47	33.1	0.179
pT4	96	35.8	58	43.1	0.652
pN stage					
pN0	37	60.9	19	63.2	0.860
pN1	81	48.8	41	51.2	0.906
pN2	92	43.1	34	53.3	0.169
pN3	77	33.9	35	16.3	0.026

n, number of patients; 5-YSR, 5-year survival rate.
